# Subject Index Volume 7

**DOI:** 10.1111/jdi.12577

**Published:** 2016-11-01

**Table:** 

25‐hydroxyvitamin D_3_
Effect of serum 25‐hydroxyvitamin D_3_ on insulin resistance and β‐cell function in newly diagnosed type 2 diabetes patients, Yang 226–232
5‐hydroxytryptamine 2 receptor antagonist
Effective treatment with combination of peripheral 5‐hydroxytryptamine synthetic inhibitor and 5‐hydroxytryptamine 2 receptor antagonist on glucocorticoid‐induced wholebody insulin resistance with hyperglycemia, Ma 833–844
5‐hydroxytryptamine synthesis
Important role of 5‐hydroxytryptamine in glucocorticoid‐induced insulin resistance in liver and intra‐abdominal adipose tissue of rats, Li 32–41
5‐hydroxytryptamine synthetic inhibitor
Effective treatment with combination of peripheral 5‐hydroxytryptamine synthetic inhibitor and 5‐hydroxytryptamine 2 receptor antagonist on glucocorticoid‐induced wholebody insulin resistance with hyperglycemia, Ma 833–844
acetyl‐L‐carnitine
Effects of acetyl‐L‐carnitine and methylcobalamin for diabetic peripheral neuropathy: A multicenter, randomized, double‐blind, controlled trial, Li 777–785
add on to insulin
Efficacy and safety of dapagliflozin in addition to insulin therapy in Japanese patients with type 2 diabetes: Results of the interim analysis of 16‐week double‐blind treatment period, Araki 555–564
adenosine monophosphate‐activated protein kinase
Diabetic complications within the context of aging: Nicotinamide adenine dinucleotide redox, insulin C‐peptide, sirtuin 1–liver kinase B1–adenosine monophosphate‐activated protein kinase positive feedback and forkhead box O3, Ido 448–458
adipocyte fatty acid binding protein
Changes in serum adipocyte fatty acid‐binding protein in women with gestational diabetes mellitus and normal pregnant women during mid‐ and late pregnancy, Zhang 797–804
adipocytokine
Changes in serum adipocyte fatty acid‐binding protein in women with gestational diabetes mellitus and normal pregnant women during mid‐ and late pregnancy, Zhang 797–804
*ADIPOQ*
Association of *ADIPOQ* variants with type 2 diabetes mellitus susceptibility in ethnic Han Chinese from northeast China, Yao 853–859
adult
Association of the hypertriglyceridemic waist phenotype and type 2 diabetes mellitus among adults in China, Ren 689–694
alpha 2‐macroglobulin
Association of salivary alpha 2‐macroglobulin levels and clinical characteristics in type 2 diabetes, Chung 190–196
anal fistula
Case of anal fistula with Fournier's gangrene in an obese type 2 diabetes mellitus patient, Yoshino 276–278
androgen deprivation therapy
Androgen deprivation therapy is associated with diabetes: Evidence from meta‐analysis, Wang 629–636
anemia
Lack of association between anemia and renal disease progression in Chinese patients with type 2 diabetes, Gu 42–47
angiopoietin‐2
Association between interleukin‐19 and angiopoietin‐2 with vascular complications in type 2 diabetes, Li 895–900
antihyperglycemic prescription
Comparison of clinical characteristics in patients with type 2 diabetes among whom different antihyperglycemic agents were prescribed as monotherapy or combination therapy by diabetes specialists, Fujihara 260–269
anti‐programmed cell death‐1 antibodies
Fulminant type 1 diabetes mellitus with anti‐programmed cell death‐1 therapy, Okamoto 915–918
artificial stiffness
Independent association between glycated hemoglobin and arterial stiffness in healthy men, Noh 241–246
Asia
Efficacy, safety, and tolerability of ipragliflozin in Asian patients with type 2 diabetes mellitus and inadequate glycemic control with metformin: Results of a phase 3 randomized, placebo‐controlled, double‐blind, multicenter trial, Lu 366–373
asymptomatic diabetes
*Klebsiella pneumoniae* thyroid abscess complicated with esophagitis in a woman with newly diagnosed diabetes mellitus: A case report, Liu 127–129
atherosclerosis
Berberine activates peroxisome proliferator‐activated receptor gamma to increase atherosclerotic plaque stability in *Apoe*‐/‐ mice with hyperhomocysteinemia, Li 824–832
atrial fibrillation
Diabetes mellitus is an independent risk factor for atrial fibrillation in a general Chinese population, Sun 791–796
autonomic function
Study on autonomic dysfunction and metabolic syndrome in Chinese patients, Zhu 901–907
β‐cell regeneration
β‐Cell regeneration through the transdifferentiation of pancreatic cells: Pancreatic progenitor cells in the pancreas, Kim 286–296
basal–bolus treatment
Efficacy and safety of insulin degludec in Japanese patients with type 1 and type 2 diabetes: 24‐week results from the observational study in routine clinical practice, Kobuke 94–99
basal energy expenditure
Fat‐free mass and calf circumference as body composition indices to determine non‐exercise activity thermogenesis in patients with diabetes, Isobe 352–358
basal insulin
Efficacy and safety of once‐daily insulin degludec dosed flexibly at convenient times vs fixed dosing at the same time each day in a Japanese cohort with type 2 diabetes: A randomized, 26‐week, treat‐to‐target trial, Kadowaki 711–717
berberine
Berberine activates peroxisome proliferator‐activated receptor gamma to increase atherosclerotic plaque stability in *Apoe*‐/‐ mice with hyperhomocysteinemia, Li 824–832
beta‐3 adrenergic receptor
Effects of lifestyle intervention on weight and metabolic parameters in patients with impaired glucose tolerance related to beta‐3 adrenergic receptor gene polymorphism Trp64Arg(C/T): Results from the Japan Diabetes Prevention Program, Sakane 338–342
bilirubin
Serum bilirubin levels are positively associated with glycemic variability in women with type 2 diabetes, Kim 874–880
biological marker
T cell immunoglobulin and mucin domain‐containing molecule 3 on CD14^+^ monocytes serves as a novel biological marker for diabetes duration in type 2 diabetes mellitus, Yan 867–873
biphasic insulin aspart
Subject‐driven titration of biphasic insulin aspart 30 twice daily is non‐inferior to investigator‐driven titration in Chinese patients with type 2 diabetes inadequately controlled with premixed human insulin: A randomized, open‐label, parallel‐group, multicenter trial, Yang 85–93
birthweight
Association between glycemic control and birthweight with glycated albumin in Chinese women with gestational diabetes mellitus, Li 48–55
body mass index
Influence of atherosclerosis‐related risk factors on serum high‐sensitivity C‐reactive protein levels in patients with type 2 diabetes: Comparison of their influence in obese and non‐obese patients, Shimoda 197–205
Efficacy and safety of ipragliflozin in Japanese patients with type 2 diabetes stratified by body mass index: A subgroup analysis of five randomized clinical trials, Kashiwagi 544–554
Optimal cut‐off levels of obesity indices by different definitions of metabolic syndrome in a southeast rural Chinese population, Pan 594–600
Increased visceral adiposity with normal weight is associated with the prevalence of non‐alcoholic fatty liver disease in Japanese patients with type 2 diabetes, Bouchi 607–614
Body mass index and the risk of cancer incidence in patients with type 2 diabetes in Japan: Results from the National Center Diabetes Database, Yamamoto‐Honda 908–914
bone marrow cell
Comparison of the effect of bone marrow cells infusion through the portal vein and inferior vena cava combined with short‐term rapamycin on allogeneic islet grafts in diabetic rats, Gao 476–484
C‐peptide
Diabetic complications within the context of aging: Nicotinamide adenine dinucleotide redox, insulin C‐peptide, sirtuin 1–liver kinase B1–adenosine monophosphate‐activated protein kinase positive feedback and forkhead box O3, Ido 448–458
calf circumference
Fat‐free mass and calf circumference as body composition indices to determine non‐exercise activity thermogenesis in patients with diabetes, Isobe 352–358
canagliflozin
Efficacy of canagliflozin combined with antidiabetic drugs in treating type 2 diabetes mellitus: Meta‐analysis of randomized control trials, Meng 359–365
cancer
Body mass index and the risk of cancer incidence in patients with type 2 diabetes in Japan: Results from the National Center Diabetes Database, Yamamoto‐Honda 908–914
cardiovascular diseases
Independent association between glycated hemoglobin and arterial stiffness in healthy men, Noh 241–246
Carpenter and Coustan criteria
Diagnosis of more gestational diabetes lead to better pregnancy outcomes: Comparing the International Association of the Diabetes and Pregnancy Study Group criteria, and the Carpenter and Coustan criteria, Wu 121–126
cell transplantation
Transplantation of dental pulp stem cells suppressed inflammation in sciatic nerves by promoting macrophage polarization towards anti‐inflammation phenotypes and ameliorated diabetic polyneuropathy, Omi 485–496
chemokine ligand 5
Association of chemokine ligand 5/chemokine receptor 5 gene promoter polymorphisms with diabetic microvascular complications: A meta‐analysis, Zhang 212–218
chemokine receptor 5
Association of chemokine ligand 5/chemokine receptor 5 gene promoter polymorphisms with diabetic microvascular complications: A meta‐analysis, Zhang 212–218
China
Prevalence of type 2 diabetes mellitus among inland residents in China (2000–2014): A meta‐analysis, Yang 845–852
Chinese adults
Ideal glycated hemoglobin cut‐off points for screening diabetes and prediabetes in a Chinese population, Liu 695–702
circulating cell‐free mitochondrial deoxyribonucleic acid
Circulating cell‐free mitochondrial deoxyribonucleic acid is increased in coronary heart disease patients with diabetes mellitus, Liu 109–114
clinical trial
Efficacy and safety of once‐daily insulin degludec dosed flexibly at convenient times vs fixed dosing at the same time each day in a Japanese cohort with type 2 diabetes: A randomized, 26‐week, treat‐to‐target trial, Kadowaki 711–717
cohort study
Dietary carbohydrate intake, presence of obesity and the incident risk of type 2 diabetes in Japanese men, Sakurai 343–351
color‐coded etiological key
Color‐coded etiological keys: A simple survey tool towards amputation‐free limb survival in diabetic foot lesions, Sharkawy 413–419
combination drug therapy
Clinical study of repaglinide efficacy and safety in type 2 diabetes mellitus patients with blood glucose levels inadequately controlled by sitagliptin, Kawamori 253–259
combination therapy
Comparison of clinical characteristics in patients with type 2 diabetes among whom different antihyperglycemic agents were prescribed as monotherapy or combination therapy by diabetes specialists, Fujihara 260–269
Commentary
A novel role for the cell cycle regulatory complex cyclin D1–CDK4 in gluconeogenesis, Hosooka 27–28
Renoprotective effects of incretin‐based drugs: A novel pleiotropic effect of dipeptidyl peptidase‐4 inhibitor, Kodera 29–31
Control of intestinal stem cell fate: A novel approach to treating diabetes, Yamane 166–168
Autophagy: Starved β‐cells seem different from starved body, Jung 169–170
Involvement of advanced glycation end‐products in ‘hyperglycemic memory’, Nishikawa 297–299
Combined intervention for the tertiary prevention of type 1 diabetes, Kawasaki 300–302
Is ghrelin a glucagon‐like peptide‐1 secretagogue?, Ueno 466–467
Prognostic impact of cardiac troponin T in patients with stable coronary artery disease and diabetes, Amano 468–469
Urinary adiponectin and progression of diabetic nephropathy in type 1 diabetes, Ha 470–471
Fetal exposure to parental smoking and the risk of type 2 diabetes: Are lifestyle‐related factors more important?, Chang 472–475
Controversy about the relationship between sulfonylurea use and cardiovascular events and mortality, Shimoda 674–676
Novel role for the CRTC2 in lipid homeostasis, Matsuzaka 677–679
Can restoring immune balance be the ultimate therapy for type 1 diabetes?, Uchigata 819–821
Role of clock genes in insulin secretion, Harada 822–823
continuous glucose monitoring
Evaluation of safety of insulin degludec on undergoing total colonoscopy using continuous glucose monitoring, Takeishi 374–380
Serum bilirubin levels are positively associated with glycemic variability in women with type 2 diabetes, Kim 874–880
coronary artery disease
Usefulness of carotid plaque (sum and maximum of plaque thickness) in combination with intima‐media thickness for the detection of coronary artery disease in asymptomatic patients with diabetes, Akazawa 396–403
coronary heart disease
Circulating cell‐free mitochondrial deoxyribonucleic acid is increased in coronary heart disease patients with diabetes mellitus, Liu 109–114
CYP2C9*3
CYP2C93 variant is associated with antidiabetes efficacy of gliclazide in Chinese type 2 diabetes patients, Zeng 764–768
dapagliflozin
Efficacy and safety of dapagliflozin in addition to insulin therapy in Japanese patients with type 2 diabetes: Results of the interim analysis of 16‐week double‐blind treatment period, Araki 555–564
degludec
Pharmacokinetic and pharmacodynamic properties of insulin degludec in Japanese patients with type 1 diabetes mellitus reflect similarities with Caucasian patients, Ikushima 270–275
dementia
Potentials of incretin‐based therapies in dementia and stroke in type 2 diabetes mellitus, Groeneveld 5–16
dental pulp stem cells
Transplantation of dental pulp stem cells suppressed inflammation in sciatic nerves by promoting macrophage polarization towards anti‐inflammation phenotypes and ameliorated diabetic polyneuropathy, Omi 485–496
diabetes
Association of salivary alpha 2‐macroglobulin levels and clinical characteristics in type 2 diabetes, Chung 190–196
New risk‐scoring system including non‐alcoholic fatty liver disease for predicting incident type 2 diabetes in East China: Shanghai Baosteel Cohort, Chen 206–211
Androgen deprivation therapy is associated with diabetes: Evidence from meta‐analysis, Wang 629–636
Nocturia and prevalence of erectile dysfunction in Japanese patients with type 2 diabetes mellitus: The Dogo Study, Furukawa 786–790
diabetes epidemiology
2015 Yutaka Seino Distinguished Leadership Award Lecture: The Japanese American Community Diabetes Study and the ‘canary in the coal mine’, Fujimoto 664–673
diabetes mellitus
Circulating cell‐free mitochondrial deoxyribonucleic acid is increased in coronary heart disease patients with diabetes mellitus, Liu 109–114
Association of chemokine ligand 5/chemokine receptor 5 gene promoter polymorphisms with diabetic microvascular complications: A meta‐analysis, Zhang 212–218
Difference between old and young adults in contribution of β‐cell function and sarcopenia in developing diabetes mellitus, Koo 233–240
Case of anal fistula with Fournier's gangrene in an obese type 2 diabetes mellitus patient, Yoshino 276–278
Diagnosis of monogenic diabetes: 10‐Year experience in a large multi‐ethnic diabetes center, Thomas 332–337
Effects of ethanol extract of propolis on histopathological changes and anti‐oxidant defense of kidney in a rat model for type 1 diabetes mellitus, Sameni 506–513
diabetes mellitus
Diabetes mellitus is an independent risk factor for atrial fibrillation in a general Chinese population, Sun 791–796
diabetes prevention
Effects of lifestyle intervention on weight and metabolic parameters in patients with impaired glucose tolerance related to beta‐3 adrenergic receptor gene polymorphism Trp64Arg(C/T): Results from the Japan Diabetes Prevention Program, Sakane 338–342
diabetes‐related complications
Efficacy and safety of 40 mg or 60 mg duloxetine in Japanese adults with diabetic neuropathic pain: Results from a randomized, 52‐week, open‐label study, Yasuda 100–108
diabetes specialists
Comparison of clinical characteristics in patients with type 2 diabetes among whom different antihyperglycemic agents were prescribed as monotherapy or combination therapy by diabetes specialists, Fujihara 260–269
diabetic complication
Assessment of the relationship between non‐alcoholic fatty liver disease and diabetic complications, Yan 889–894
diabetic complications
Genetics of cardiovascular and renal complications in diabetes, Ma 139–154
diabetic ketoacidosis
Case of disseminated pyomyositis in poorly controlled type 2 diabetes mellitus with diabetic ketoacidosis, Tanabe 637–640
diabetic kidney disease
Genetics of cardiovascular and renal complications in diabetes, Ma 139–154
diabetic neuropathy
Elasticity of the tibial nerve assessed by sonoelastography was reduced before the development of neuropathy and further deterioration associated with the severity of neuropathy in patients with type 2 diabetes, Ishibashi 404–412
Retrograde pyelonephritis and lumbar spondylitis as a result of *Salmonella typhi* in a type 2 diabetes patient with neurogenic bladder, Fukuda 436–439
diabetic neuropathy (painful)
Efficacy and safety of 40 mg or 60 mg duloxetine in Japanese adults with diabetic neuropathic pain: Results from a randomized, 52‐week, open‐label study, Yasuda 100–108
diabetic peripheral neuropathy
Effects of acetyl‐L‐carnitine and methylcobalamin for diabetic peripheral neuropathy: A multicenter, randomized, double‐blind, controlled trial, Li 777–785
diabetic polyneuropathy
Transplantation of dental pulp stem cells suppressed inflammation in sciatic nerves by promoting macrophage polarization towards anti‐inflammation phenotypes and ameliorated diabetic polyneuropathy, Omi 485–496
dietary
Dietary and physical activity of adult patients with type 2 diabetes in Zhejiang province of eastern China: Data from a cross‐sectional study, He 529–538
dietary carbohydrates
Dietary carbohydrate intake, presence of obesity and the incident risk of type 2 diabetes in Japanese men, Sakurai 343–351
diet‐induced thermogenesis
Fat‐free mass and calf circumference as body composition indices to determine non‐exercise activity thermogenesis in patients with diabetes, Isobe 352–358
dipeptidyl peptidase‐4 inhibitor
Randomized clinical trial of the safety and efficacy of sitagliptin and metformin co‐administered to Chinese patients with type 2 diabetes mellitus, Ji 727–736
dipeptidyl peptidase‐4 inhibitors
Adherence to dipeptidyl peptidase‐4 inhibitor therapy among type 2 diabetes patients with employer‐sponsored health insurance in Japan, Kurtyka 737–743
disease management
Cluster‐randomized trial to improve the quality of diabetes management: The study for the efficacy assessment of the standard diabetes manual (SEAS‐DM), Noto 539–543
duloxetine
Efficacy and safety of 40 mg or 60 mg duloxetine in Japanese adults with diabetic neuropathic pain: Results from a randomized, 52‐week, open‐label study, Yasuda 100–108
Editorial
Like a snail, our journal (JDI) makes slow, but steady, progress, Hotta 3–4
Euglycemic diabetic ketoacidosis induced by SGLT2 inhibitors: possible mechanism and contributing factors, Ogawa 135–138
Type 2 diabetes mellitus prevention strategy: Target and goal in Japanese men, Iwahashi 283–285
Re‐examining the high‐density lipoprotein hypothesis, Tan 445–447
Diabetes and shoulder disorders, Hsu 649–651
Pitfalls of tightening driving regulations for diabetic patients, Nakashima 809–811
elasticity of tibial nerve
Elasticity of the tibial nerve assessed by sonoelastography was reduced before the development of neuropathy and further deterioration associated with the severity of neuropathy in patients with type 2 diabetes, Ishibashi 404–412
elderly
Japanese study of tofogliflozin with type 2 diabetes mellitus patients in an observational study of the elderly (J‐STEP/EL): A 12‐week interim analysis, Utsunomiya 755–763
endoplasmic reticulum stress
Endoplasmic reticulum stress induced by tunicamycin increases resistin messenger ribonucleic acid through the pancreatic endoplasmic reticulum eukaryotic initiation factor 2α kinase–activating transcription factor 4–CAAT/enhancer binding protein‐α homologous protein pathway in THP‐1 human monocytes, Hamada 312–323
erectile dysfunction
Nocturia and prevalence of erectile dysfunction in Japanese patients with type 2 diabetes mellitus: The Dogo Study, Furukawa 786–790
esophagitis
*Klebsiella pneumoniae* thyroid abscess complicated with esophagitis in a woman with newly diagnosed diabetes mellitus: A case report, Liu 127–129
etiological factors in Asians
2015 Yutaka Seino Distinguished Leadership Award Lecture: The Japanese American Community Diabetes Study and the ‘canary in the coal mine’, Fujimoto 664–673
exercise
Cardiovascular autonomic neuropathy in patients with type 2 diabetes, Liu 615–621
exposure–response
Comparable pharmacodynamics, efficacy, and safety of linagliptin 5 mg among Japanese, Asian and white patients with type 2 diabetes, Sarashina 744–750
Fournier's gangrene
Case of anal fistula with Fournier's gangrene in an obese type 2 diabetes mellitus patient, Yoshino 276–278
fulminant type 1 diabetes
Fulminant type 1 diabetes mellitus with anti‐programmed cell death‐1 therapy, Okamoto 915–918
genetics
Genetics of cardiovascular and renal complications in diabetes, Ma 139–154
HLA‐A*33‐DR3 and A*33‐DR9 haplotypes enhance the risk of type 1 diabetes in Han Chinese, Zhang 514–521
genetic testing
Diagnosis of monogenic diabetes: 10‐Year experience in a large multi‐ethnic diabetes center, Thomas 332–337
gestational diabetes
Association between glycemic control and birthweight with glycated albumin in Chinese women with gestational diabetes mellitus, Li 48–55
Diagnosis of more gestational diabetes lead to better pregnancy outcomes: Comparing the International Association of the Diabetes and Pregnancy Study Group criteria, and the Carpenter and Coustan criteria, Wu 121–126
gestational diabetes mellitus
Correlations of serum visfatin and metabolisms of glucose and lipid in women with gestational diabetes mellitus, Liang 247–252
Serum homocysteine level and gestational diabetes mellitus: A meta‐analysis, Gong 622–628
Changes in serum adipocyte fatty acid‐binding protein in women with gestational diabetes mellitus and normal pregnant women during mid‐ and late pregnancy, Zhang 797–804
ghrelin
Phenotyping of type 2 diabetes mellitus at onset on the basis of fasting incretin tone: Results of a two‐step cluster analysis, Amato 219–225
gliclazide
CYP2C93 variant is associated with antidiabetes efficacy of gliclazide in Chinese type 2 diabetes patients, Zeng 764–768
glucagon‐like peptide‐1
Phenotyping of type 2 diabetes mellitus at onset on the basis of fasting incretin tone: Results of a two‐step cluster analysis, Amato 219–225
glucocorticoid
Important role of 5‐hydroxytryptamine in glucocorticoid‐induced insulin resistance in liver and intra‐abdominal adipose tissue of rats, Li 32–41
glucocorticoid‐induced insulin resistance
Effective treatment with combination of peripheral 5‐hydroxytryptamine synthetic inhibitor and 5‐hydroxytryptamine 2 receptor antagonist on glucocorticoid‐induced wholebody insulin resistance with hyperglycemia, Ma 833–844
glucagon (1–29)
Postabsorptive hyperglucagonemia in patients with type 2 diabetes mellitus analyzed with a novel enzyme‐linked immunosorbent assay, Matsuo 324–331
glucagon‐like peptide‐1 receptor agonists
Combination therapy of glucagon‐like peptide‐1 receptor agonists and insulin for patients who developed diabetes after partial pancreatectomy, Kitazawa 381–385
glucagon‐like peptides
Postabsorptive hyperglucagonemia in patients with type 2 diabetes mellitus analyzed with a novel enzyme‐linked immunosorbent assay, Matsuo 324–331
glucokinase
Src regulates insulin secretion and glucose metabolism by influencing subcellular localization of glucokinase in pancreatic β‐cells, Sato 171–178
glucose and lipid metabolism
Expression profiling analysis: Uncoupling protein 2 deficiency improves hepatic glucose, lipid profiles and insulin sensitivity in high‐fat diet‐fed mice by modulating expression of genes in peroxisome proliferator‐activated receptor signaling pathway, Zhou 179–189
Correlations of serum visfatin and metabolisms of glucose and lipid in women with gestational diabetes mellitus, Liang 247–252
glucose‐dependent insulinotropic polypeptide
Phenotyping of type 2 diabetes mellitus at onset on the basis of fasting incretin tone: Results of a two‐step cluster analysis, Amato 219–225
Anti‐inflammatory role of glucose‐dependent insulinotropic polypeptide in periodontitis, Suzuki 497–505
glucose metabolism
Low osteocalcin level is a risk factor for impaired glucose metabolism in a Chinese male population, Liang 522–528
glucose variability
Serum bilirubin levels are positively associated with glycemic variability in women with type 2 diabetes, Kim 874–880
glycated albumin
Association between glycemic control and birthweight with glycated albumin in Chinese women with gestational diabetes mellitus, Li 48–55
glycated hemoglobin
Independent association between glycated hemoglobin and arterial stiffness in healthy men, Noh 241–246
glycemic control
Randomized clinical trial of the safety and efficacy of sitagliptin and metformin co‐administered to Chinese patients with type 2 diabetes mellitus, Ji 727–736
Pitavastatin improves glycated hemoglobin in patients with poorly controlled type 2 diabetes, Huang 769–776
glycemic variability
Hypoglycemia and glycemic variability are associated with mortality in non‐intensive care unit hospitalized infectious disease patients with diabetes mellitus, Takeishi 429–435
gut peptides
Mechanistic relationship between the vagal afferent pathway, central nervous system and peripheral organs in appetite regulation, Ueno 812–818
heart rate recovery
Cardiovascular autonomic neuropathy in patients with type 2 diabetes, Liu 615–621
hematuria
Dysmorphic erythrocytes are superior to hematuria for indicating non‐diabetic renal disease in type 2 diabetics, Dong 115–120
hepatocyte nuclear factor 1‐alpha
Diagnosis of monogenic diabetes: 10‐Year experience in a large multi‐ethnic diabetes center, Thomas 332–337
high‐sensitivity C‐reactive protein
Influence of atherosclerosis‐related risk factors on serum high‐sensitivity C‐reactive protein levels in patients with type 2 diabetes: Comparison of their influence in obese and non‐obese patients, Shimoda 197–205
HLA‐A*33
HLA‐A*33‐DR3 and A*33‐DR9 haplotypes enhance the risk of type 1 diabetes in Han Chinese, Zhang 514–521
homocysteine
Serum homocysteine level and gestational diabetes mellitus: A meta‐analysis, Gong 622–628
human monocytes
Endoplasmic reticulum stress induced by tunicamycin increases resistin messenger ribonucleic acid through the pancreatic endoplasmic reticulum eukaryotic initiation factor 2α kinase–activating transcription factor 4–CAAT/enhancer binding protein‐α homologous protein pathway in THP‐1 human monocytes, Hamada 312–323
hyperhomocysteinemia
Serum homocysteine level and gestational diabetes mellitus: A meta‐analysis, Gong 622–628
hypertension
Diabetes mellitus is an independent risk factor for atrial fibrillation in a general Chinese population, Sun 791–796
hypertriglyceridemic waist
Utility of hypertriglyceridemic waist phenotype for predicting incident type 2 diabetes: The Isfahan Diabetes Prevention Study, Janghorbani 860–866
hypertriglyceridemic waist phenotype
Association of the hypertriglyceridemic waist phenotype and type 2 diabetes mellitus among adults in China, Ren 689–694
hypoglycemia
Efficacy and safety of insulin degludec in Japanese patients with type 1 and type 2 diabetes: 24‐week results from the observational study in routine clinical practice, Kobuke 94–99
Hypoglycemia and glycemic variability are associated with mortality in non‐intensive care unit hospitalized infectious disease patients with diabetes mellitus, Takeishi 429–435
hypothalamus
Mechanistic relationship between the vagal afferent pathway, central nervous system and peripheral organs in appetite regulation, Ueno 812–818
immune cells
Neutrophils in type 1 diabetes, Huang 652–663
impaired glucose tolerance
Effects of lifestyle intervention on weight and metabolic parameters in patients with impaired glucose tolerance related to beta‐3 adrenergic receptor gene polymorphism Trp64Arg(C/T): Results from the Japan Diabetes Prevention Program, Sakane 338–342
Elevated circulating level of a cytokine, pancreatic‐derived factor, is associated with metabolic syndrome components in a Chinese population, Cao 581–586
incidence
Dietary carbohydrate intake, presence of obesity and the incident risk of type 2 diabetes in Japanese men, Sakurai 343–351
incretins
Potentials of incretin‐based therapies in dementia and stroke in type 2 diabetes mellitus, Groeneveld 5–16
incretin therapy
Randomized clinical trial of the safety and efficacy of sitagliptin and metformin co‐administered to Chinese patients with type 2 diabetes mellitus, Ji 727–736
inflammation
Anti‐inflammatory role of glucose‐dependent insulinotropic polypeptide in periodontitis, Suzuki 497–505
insulin
Pharmacokinetic and pharmacodynamic properties of insulin degludec in Japanese patients with type 1 diabetes mellitus reflect similarities with Caucasian patients, Ikushima 270–275
Combination therapy with liraglutide and insulin in Japanese patients with type 2 diabetes: A 36‐week, randomized, double‐blind, parallel‐group trial, Seino 565–573
insulin degludec
Efficacy and safety of insulin degludec in Japanese patients with type 1 and type 2 diabetes: 24‐week results from the observational study in routine clinical practice, Kobuke 94–99
Evaluation of safety of insulin degludec on undergoing total colonoscopy using continuous glucose monitoring, Takeishi 374–380
A 1‐year, prospective, observational study of Japanese outpatients with type 1 and type 2 diabetes switching from insulin glargine or detemir to insulin degludec in basal–bolus insulin therapy (Kumamoto Insulin Degludec Observational study), Shimoda 703–710
insulin degludec – insulin aspart
Insulin degludec/insulin aspart in Japanese patients with type 1 diabetes mellitus: Distinct prandial and basal glucose‐lowering effects, Haahr 574–580
insulin detemir
A 1‐year, prospective, observational study of Japanese outpatients with type 1 and type 2 diabetes switching from insulin glargine or detemir to insulin degludec in basal–bolus insulin therapy (Kumamoto Insulin Degludec Observational study), Shimoda 703–710
insulin glargine
A 1‐year, prospective, observational study of Japanese outpatients with type 1 and type 2 diabetes switching from insulin glargine or detemir to insulin degludec in basal–bolus insulin therapy (Kumamoto Insulin Degludec Observational study), Shimoda 703–710
insulin resistance
Important role of 5‐hydroxytryptamine in glucocorticoid‐induced insulin resistance in liver and intra‐abdominal adipose tissue of rats, Li 32–41
Effect of serum 25‐hydroxyvitamin D_3_ on insulin resistance and β‐cell function in newly diagnosed type 2 diabetes patients, Yang 226–232
Low osteocalcin level is a risk factor for impaired glucose metabolism in a Chinese male population, Liang 522–528
insulin secretion
Src regulates insulin secretion and glucose metabolism by influencing subcellular localization of glucokinase in pancreatic β‐cells, Sato 171–178
insulin therapy
Glycemic control and adherence to basal insulin therapy in Taiwanese patients with type 2 diabetes mellitus, Chien 881–888
interleukin‐19
Association between interleukin‐19 and angiopoietin‐2 with vascular complications in type 2 diabetes, Li 895–900
International Association of the Diabetes and Pregnancy Study Group criteria
Diagnosis of more gestational diabetes lead to better pregnancy outcomes: Comparing the International Association of the Diabetes and Pregnancy Study Group criteria, and the Carpenter and Coustan criteria, Wu 121–126
ipragliflozin
Efficacy, safety, and tolerability of ipragliflozin in Asian patients with type 2 diabetes mellitus and inadequate glycemic control with metformin: Results of a phase 3 randomized, placebo‐controlled, double‐blind, multicenter trial, Lu 366–373
Efficacy and safety of ipragliflozin in Japanese patients with type 2 diabetes stratified by body mass index: A subgroup analysis of five randomized clinical trials, Kashiwagi 544–554
iridocyclitis
Syphilis iridocyclitis in a patient with type 1 diabetes, Comassi 641–644
islet
Dynamic pathology of islet endocrine cells in type 2 diabetes: β‐Cell growth, death, regeneration and their clinical implications, Yagihashi 155–165
islet grafts
Comparison of the effect of bone marrow cells infusion through the portal vein and inferior vena cava combined with short‐term rapamycin on allogeneic islet grafts in diabetic rats, Gao 476–484
Japanese
Pharmacokinetic and pharmacodynamic properties of insulin degludec in Japanese patients with type 1 diabetes mellitus reflect similarities with Caucasian patients, Ikushima 270–275
Insulin degludec/insulin aspart in Japanese patients with type 1 diabetes mellitus: Distinct prandial and basal glucose‐lowering effects, Haahr 574–580
Klotho
Periodontitis aggravated pancreatic β‐cell dysfunction in diabetic mice through interleukin‐12 regulation on Klotho, Liu 303–311
Letter to the Editor
Osmotic demyelination syndrome complicating diabetes with anti‐glutamic acid decarboxylase antibodies and Graves’ disease: A case report, Tajitsu 130–131
Case of iliopsoas abscess that was markedly recovered after percutaneous and surgical drainage in a patient with poorly controlled type 2 diabetes, Obata 440–441
Optimal cut‐off value of alanine aminotransferase level to precisely estimate the presence of fatty liver in patients with poorly controlled type 2 diabetes, Tanabe 645–646
Improvement of glycemic control without severe hypoglycemia in a type 1 diabetes patient undergoing hemodialysis after a change from insulin glargine to insulin degludec, Takahashi 805–806
Human serum albumin: Possible cause of insulin autoimmune syndrome, Kamei 919–920
lifestyle
Dietary and physical activity of adult patients with type 2 diabetes in Zhejiang province of eastern China: Data from a cross‐sectional study, He 529–538
linagliptin
Comparable pharmacodynamics, efficacy, and safety of linagliptin 5 mg among Japanese, Asian and white patients with type 2 diabetes, Sarashina 744–750
liraglutide
Liraglutide is effective and well tolerated in combination with an oral antidiabetic drug in Japanese patients with type 2 diabetes: A randomized, 52‐week, open‐label, parallel‐group trial Kaku 76–84
Combination therapy with liraglutide and insulin in Japanese patients with type 2 diabetes: A 36‐week, randomized, double‐blind, parallel‐group trial, Seino 565–573
lixisenatide
Combination therapy of glucagon‐like peptide‐1 receptor agonists and insulin for patients who developed diabetes after partial pancreatectomy, Kitazawa 381–385
long‐term safety and efficacy
Long‐term safety and efficacy of a novel once‐weekly oral trelagliptin as monotherapy or in combination with an existing oral antidiabetic drug in patients with type 2 diabetes mellitus: A 52‐week open‐label, phase 3 study, Inagaki 718–726
maximal plaque thickness
Usefulness of carotid plaque (sum and maximum of plaque thickness) in combination with intima‐media thickness for the detection of coronary artery disease in asymptomatic patients with diabetes, Akazawa 396–403
medication adherence
Adherence to dipeptidyl peptidase‐4 inhibitor therapy among type 2 diabetes patients with employer‐sponsored health insurance in Japan, Kurtyka 737–743
meta‐analysis
Higher intake of fruits, vegetables or their fiber reduces the risk of type 2 diabetes: A meta‐analysis, Wang 56–69
Efficacy of canagliflozin combined with antidiabetic drugs in treating type 2 diabetes mellitus: Meta‐analysis of randomized control trials, Meng 359–365
Androgen deprivation therapy is associated with diabetes: Evidence from meta‐analysis, Wang 629–636
metabolic parameters
Metabolic parameters in type 2 diabetic patients with varying degrees of glycemic control during Ramadan: An observational study, Siaw 70–75
metabolic risk factors
Associations between metabolic risk factors and body mass index, waist circumference, waist‐to‐height ratio and waist‐to‐hip ratio in a Chinese rural population, Guan 601–606
metabolic syndrome
Elevated circulating level of a cytokine, pancreatic‐derived factor, is associated with metabolic syndrome components in a Chinese population, Cao 581–586
Proposed cut‐off values of the waist circumference for metabolic syndrome based on visceral fat volume in a Japanese population, Tsukiyama 587–593
Optimal cut‐off levels of obesity indices by different definitions of metabolic syndrome in a southeast rural Chinese population, Pan 594–600
Study on autonomic dysfunction and metabolic syndrome in Chinese patients, Zhu 901–907
methylcobalamin
Effects of acetyl‐L‐carnitine and methylcobalamin for diabetic peripheral neuropathy: A multicenter, randomized, double‐blind, controlled trial, Li 777–785
microglia
Microglia in the spinal cord and neuropathic pain, Tsuda 17–26
mortality
Hypoglycemia and glycemic variability are associated with mortality in non‐intensive care unit hospitalized infectious disease patients with diabetes mellitus, Takeishi 429–435
multiple intramuscular abscess
Case of disseminated pyomyositis in poorly controlled type 2 diabetes mellitus with diabetic ketoacidosis, Tanabe 637–640
nephropathy
Dysmorphic erythrocytes are superior to hematuria for indicating non‐diabetic renal disease in type 2 diabetics, Dong 115–120
Vitamin D and its receptor regulate lipopolysaccharide‐induced transforming growth factor‐β, angiotensinogen expression and podocytes apoptosis through the nuclear factor‐κB pathway, Xu 680–688
neutrophils
Neutrophils in type 1 diabetes, Huang 652–663
newly diagnosed type 2 diabetes
Effect of serum 25‐hydroxyvitamin D_3_ on insulin resistance and β‐cell function in newly diagnosed type 2 diabetes patients, Yang 226–232
nivolumab
Fulminant type 1 diabetes mellitus with anti‐programmed cell death‐1 therapy, Okamoto 915–918
nocturia
Nocturia and prevalence of erectile dysfunction in Japanese patients with type 2 diabetes mellitus: The Dogo Study, Furukawa 786–790
non‐alcoholic fatty liver disease
New risk‐scoring system including non‐alcoholic fatty liver disease for predicting incident type 2 diabetes in East China: Shanghai Baosteel Cohort, Chen 206–211
Increased visceral adiposity with normal weight is associated with the prevalence of non‐alcoholic fatty liver disease in Japanese patients with type 2 diabetes, Bouchi 607–614
Assessment of the relationship between non‐alcoholic fatty liver disease and diabetic complications, Yan 889–894
non‐traumatic limb amputations
Color‐coded etiological keys: A simple survey tool towards amputation‐free limb survival in diabetic foot lesions, Sharkawy 413–419
nutrition intake
Higher intake of fruits, vegetables or their fiber reduces the risk of type 2 diabetes: A meta‐analysis, Wang 56–69
observational study
Glycemic control and adherence to basal insulin therapy in Taiwanese patients with type 2 diabetes mellitus, Chien 881–888
oral antidiabetic drug
Liraglutide is effective and well tolerated in combination with an oral antidiabetic drug in Japanese patients with type 2 diabetes: A randomized, 52‐week, open‐label, parallel‐group trial Kaku 76–84
Relationship between the efficacy of oral antidiabetic drugs and clinical features in type 2 diabetic patients (JDDM38), Kanatsuka 386–395
osteocalcin
Low osteocalcin level is a risk factor for impaired glucose metabolism in a Chinese male population, Liang 522–528
oxidative stress
Effects of ethanol extract of propolis on histopathological changes and anti‐oxidant defense of kidney in a rat model for type 1 diabetes mellitus, Sameni 506–513
painful diabetic neuropathy
Microglia in the spinal cord and neuropathic pain, Tsuda 17–26
pancreatectomy
Combination therapy of glucagon‐like peptide‐1 receptor agonists and insulin for patients who developed diabetes after partial pancreatectomy, Kitazawa 381–385
pancreatic β‐cell
Periodontitis aggravated pancreatic β‐cell dysfunction in diabetic mice through interleukin‐12 regulation on Klotho, Liu 303–311
pancreatic β‐cells
Difference between old and young adults in contribution of β‐cell function and sarcopenia in developing diabetes mellitus, Koo 233–240
pancreatic‐derived factor
Elevated circulating level of a cytokine, pancreatic‐derived factor, is associated with metabolic syndrome components in a Chinese population, Cao 581–586
pancreatic progenitor cells
β‐Cell regeneration through the transdifferentiation of pancreatic cells: Pancreatic progenitor cells in the pancreas, Kim 286–296
pathology
Dynamic pathology of islet endocrine cells in type 2 diabetes: β‐Cell growth, death, regeneration and their clinical implications, Yagihashi 155–165
pathophysiology in Asians
2015 Yutaka Seino Distinguished Leadership Award Lecture: The Japanese American Community Diabetes Study and the ‘canary in the coal mine’, Fujimoto 664–673
periodontal disease
Anti‐inflammatory role of glucose‐dependent insulinotropic polypeptide in periodontitis, Suzuki 497–505
periodontitis
Periodontitis aggravated pancreatic β‐cell dysfunction in diabetic mice through interleukin‐12 regulation on Klotho, Liu 303–311
peroxisome proliferator‐activated receptor‐γ
Berberine activates peroxisome proliferator‐activated receptor gamma to increase atherosclerotic plaque stability in *Apoe*‐/‐ mice with hyperhomocysteinemia, Li 824–832
peroxisome proliferator‐activated receptor signaling pathway
Expression profiling analysis: Uncoupling protein 2 deficiency improves hepatic glucose, lipid profiles and insulin sensitivity in high‐fat diet‐fed mice by modulating expression of genes in peroxisome proliferator‐activated receptor signaling pathway, Zhou 179–189
pharmacodynamics
Insulin degludec/insulin aspart in Japanese patients with type 1 diabetes mellitus: Distinct prandial and basal glucose‐lowering effects, Haahr 574–580
plaque score
Usefulness of carotid plaque (sum and maximum of plaque thickness) in combination with intima‐media thickness for the detection of coronary artery disease in asymptomatic patients with diabetes, Akazawa 396–403
podocyte
Vitamin D and its receptor regulate lipopolysaccharide‐induced transforming growth factor‐β, angiotensinogen expression and podocytes apoptosis through the nuclear factor‐κB pathway, Xu 680–688
prevalence
Prevalence of type 2 diabetes mellitus among inland residents in China (2000–2014): A meta‐analysis, Yang 845–852
propensity score‐matched cohort study
Relationship between the efficacy of oral antidiabetic drugs and clinical features in type 2 diabetic patients (JDDM38), Kanatsuka 386–395
propolis
Effects of ethanol extract of propolis on histopathological changes and anti‐oxidant defense of kidney in a rat model for type 1 diabetes mellitus, Sameni 506–513
proportional hazard models
New risk‐scoring system including non‐alcoholic fatty liver disease for predicting incident type 2 diabetes in East China: Shanghai Baosteel Cohort, Chen 206–211
psychological impact
Psychological impacts from expectation of worsening conditions and obstacles to life planning are affected by glycemic control, self‐reported symptoms, and drug therapy in patients with type 2 diabetes mellitus, Nakao 420–428
purinergic receptors
Microglia in the spinal cord and neuropathic pain, Tsuda 17–26
pyomyositis
Case of disseminated pyomyositis in poorly controlled type 2 diabetes mellitus with diabetic ketoacidosis, Tanabe 637–640
quality indicator
Cluster‐randomized trial to improve the quality of diabetes management: The study for the efficacy assessment of the standard diabetes manual (SEAS‐DM), Noto 539–543
race effect
Comparable pharmacodynamics, efficacy, and safety of linagliptin 5 mg among Japanese, Asian and white patients with type 2 diabetes, Sarashina 744–750
Ramadan
Metabolic parameters in type 2 diabetic patients with varying degrees of glycemic control during Ramadan: An observational study, Siaw 70–75
randomized trial
Cluster‐randomized trial to improve the quality of diabetes management: The study for the efficacy assessment of the standard diabetes manual (SEAS‐DM), Noto 539–543
rapamycin
Comparison of the effect of bone marrow cells infusion through the portal vein and inferior vena cava combined with short‐term rapamycin on allogeneic islet grafts in diabetic rats, Gao 476–484
receiver operating characteristic curves
Ideal glycated hemoglobin cut‐off points for screening diabetes and prediabetes in a Chinese population, Liu 695–702
renal disease progression
Lack of association between anemia and renal disease progression in Chinese patients with type 2 diabetes, Liubao Gu 42–47
renal threshold for glucose reabsorption
Renal threshold for glucose reabsorption predicts diabetes improvement by sodium‐glucose cotransporter 2 inhibitor therapy, Osaki 751–754
repaglinide
Clinical study of repaglinide efficacy and safety in type 2 diabetes mellitus patients with blood glucose levels inadequately controlled by sitagliptin, Kawamori 253–259
resistin
Endoplasmic reticulum stress induced by tunicamycin increases resistin messenger ribonucleic acid through the pancreatic endoplasmic reticulum eukaryotic initiation factor 2α kinase–activating transcription factor 4–CAAT/enhancer binding protein‐α homologous protein pathway in THP‐1 human monocytes, Hamada 312–323
retrograde pyelonephritis
Retrograde pyelonephritis and lumbar spondylitis as a result of *Salmonella typhi* in a type 2 diabetes patient with neurogenic bladder, Fukuda 436–439
risk factor
Utility of hypertriglyceridemic waist phenotype for predicting incident type 2 diabetes: The Isfahan Diabetes Prevention Study, Janghorbani 860–866
saliva
Association of salivary alpha 2‐macroglobulin levels and clinical characteristics in type 2 diabetes, Chung 190–196
*Salmonella typhi*
Retrograde pyelonephritis and lumbar spondylitis as a result of *Salmonella typhi* in a type 2 diabetes patient with neurogenic bladder, Fukuda 436–439
sarcopenia
Difference between old and young adults in contribution of β‐cell function and sarcopenia in developing diabetes mellitus, Koo 233–240
screening and management of diabetic foot patients
Color‐coded etiological keys: A simple survey tool towards amputation‐free limb survival in diabetic foot lesions, Sharkawy 413–419
single nucleotide polymorphism
Association of *ADIPOQ* variants with type 2 diabetes mellitus susceptibility in ethnic Han Chinese from northeast China, Yao 853–859
sirtuin 1
Diabetic complications within the context of aging: Nicotinamide adenine dinucleotide redox, insulin C‐peptide, sirtuin 1–liver kinase B1–adenosine monophosphate‐activated protein kinase positive feedback and forkhead box O3, Ido 448–458
sitagliptin
Clinical study of repaglinide efficacy and safety in type 2 diabetes mellitus patients with blood glucose levels inadequately controlled by sitagliptin, Kawamori 253–259
sodium‐glucose co‐transporter 2
Efficacy and safety of ipragliflozin in Japanese patients with type 2 diabetes stratified by body mass index: A subgroup analysis of five randomized clinical trials, Kashiwagi 544–554
Efficacy and safety of dapagliflozin in addition to insulin therapy in Japanese patients with type 2 diabetes: Results of the interim analysis of 16‐week double‐blind treatment period, Araki 555–564
sodium‐glucose cotransporter 2 inhibitor
Renal threshold for glucose reabsorption predicts diabetes improvement by sodium‐glucose cotransporter 2 inhibitor therapy, Osaki 751–754
sodium‐glucose transporter 2
Japanese study of tofogliflozin with type 2 diabetes mellitus patients in an observational study of the elderly (J‐STEP/EL): A 12‐week interim analysis, Utsunomiya 755–763
sonoelastography
Elasticity of the tibial nerve assessed by sonoelastography was reduced before the development of neuropathy and further deterioration associated with the severity of neuropathy in patients with type 2 diabetes, Ishibashi 404–412
Src
Src regulates insulin secretion and glucose metabolism by influencing subcellular localization of glucokinase in pancreatic β‐cells, Sato 171–178
statin
Pitavastatin improves glycated hemoglobin in patients with poorly controlled type 2 diabetes, Huang 769–776
stroke
Potentials of incretin‐based therapies in dementia and stroke in type 2 diabetes mellitus, Groeneveld 5–16
subjective symptoms
Psychological impacts from expectation of worsening conditions and obstacles to life planning are affected by glycemic control, self‐reported symptoms, and drug therapy in patients with type 2 diabetes mellitus, Nakao 420–428
sudomotor
Study on autonomic dysfunction and metabolic syndrome in Chinese patients, Zhu 901–907
syphilis
Syphilis iridocyclitis in a patient with type 1 diabetes, Comassi 641–644
T cell immunoglobulin and mucin domain‐containing molecule 3
T cell immunoglobulin and mucin domain‐containing molecule 3 on CD14^+^ monocytes serves as a novel biological marker for diabetes duration in type 2 diabetes mellitus, Yan 867–873
thyroid abscess
*Klebsiella pneumoniae* thyroid abscess complicated with esophagitis in a woman with newly diagnosed diabetes mellitus: A case report, Liu 127–129
titration
Subject‐driven titration of biphasic insulin aspart 30 twice daily is non‐inferior to investigator‐driven titration in Chinese patients with type 2 diabetes inadequately controlled with premixed human insulin: A randomized, open‐label, parallel‐group, multicenter trial, Yang 85–93
total colonoscopy
Evaluation of safety of insulin degludec on undergoing total colonoscopy using continuous glucose monitoring, Takeishi 374–380
transdifferentiation
β‐Cell regeneration through the transdifferentiation of pancreatic cells: Pancreatic progenitor cells in the pancreas, Kim 286–296
trelagliptin
Long‐term safety and efficacy of a novel once‐weekly oral trelagliptin as monotherapy or in combination with an existing oral antidiabetic drug in patients with type 2 diabetes mellitus: A 52‐week open‐label, phase 3 study, Inagaki 718–726
type 1 diabetes
HLA‐A*33‐DR3 and A*33‐DR9 haplotypes enhance the risk of type 1 diabetes in Han Chinese, Zhang 514–521
Syphilis iridocyclitis in a patient with type 1 diabetes, Comassi 641–644
Neutrophils in type 1 diabetes, Huang 652–663
type 1 diabetes mellitus
Different role of zinc transporter 8 between type 1 diabetes mellitus and type 2 diabetes mellitus, Yi 459–465
type 2 diabetes
Metabolic parameters in type 2 diabetic patients with varying degrees of glycemic control during Ramadan: An observational study, Siaw 70–75
Subject‐driven titration of biphasic insulin aspart 30 twice daily is non‐inferior to investigator‐driven titration in Chinese patients with type 2 diabetes inadequately controlled with premixed human insulin: A randomized, open‐label, parallel‐group, multicenter trial, Yang 85–93
Dynamic pathology of islet endocrine cells in type 2 diabetes: β‐Cell growth, death, regeneration and their clinical implications, Yagihashi 155–165
Influence of atherosclerosis‐related risk factors on serum high‐sensitivity C‐reactive protein levels in patients with type 2 diabetes: Comparison of their influence in obese and non‐obese patients, Shimoda 197–205
Dietary and physical activity of adult patients with type 2 diabetes in Zhejiang province of eastern China: Data from a cross‐sectional study, He 529–538
Cardiovascular autonomic neuropathy in patients with type 2 diabetes, Liu 615–621
Adherence to dipeptidyl peptidase‐4 inhibitor therapy among type 2 diabetes patients with employer‐sponsored health insurance in Japan, Kurtyka 737–743
Utility of hypertriglyceridemic waist phenotype for predicting incident type 2 diabetes: The Isfahan Diabetes Prevention Study, Janghorbani 860–866
T cell immunoglobulin and mucin domain‐containing molecule 3 on CD14^+^ monocytes serves as a novel biological marker for diabetes duration in type 2 diabetes mellitus, Yan 867–873
Assessment of the relationship between non‐alcoholic fatty liver disease and diabetic complications, Yan 889–894
Body mass index and the risk of cancer incidence in patients with type 2 diabetes in Japan: Results from the National Center Diabetes Database, Yamamoto‐Honda 908–914
type 2 diabetes mellitus
Lack of association between anemia and renal disease progression in Chinese patients with type 2 diabetes, Gu 42–47
Higher intake of fruits, vegetables or their fiber reduces the risk of type 2 diabetes: A meta‐analysis, Wang 56–69
Liraglutide is effective and well tolerated in combination with an oral antidiabetic drug in Japanese patients with type 2 diabetes: A randomized, 52‐week, open‐label, parallel‐group trial, Kaku 76–84
Postabsorptive hyperglucagonemia in patients with type 2 diabetes mellitus analyzed with a novel enzyme‐linked immunosorbent assay, Matsuo 324–331
Efficacy of canagliflozin combined with antidiabetic drugs in treating type 2 diabetes mellitus: Meta‐analysis of randomized control trials, Meng 359–365
Efficacy, safety, and tolerability of ipragliflozin in Asian patients with type 2 diabetes mellitus and inadequate glycemic control with metformin: Results of a phase 3 randomized, placebo‐controlled, double‐blind, multicenter trial, Lu 366–373
Relationship between the efficacy of oral antidiabetic drugs and clinical features in type 2 diabetic patients (JDDM38), Kanatsuka 386–395
Psychological impacts from expectation of worsening conditions and obstacles to life planning are affected by glycemic control, self‐reported symptoms, and drug therapy in patients with type 2 diabetes mellitus, Nakao 420–428
Different role of zinc transporter 8 between type 1 diabetes mellitus and type 2 diabetes mellitus, Yi 459–465
Combination therapy with liraglutide and insulin in Japanese patients with type 2 diabetes: A 36‐week, randomized, double‐blind, parallel‐group trial, Seino 565–573
Association of the hypertriglyceridemic waist phenotype and type 2 diabetes mellitus among adults in China, Ren 689–694
Ideal glycated hemoglobin cut‐off points for screening diabetes and prediabetes in a Chinese population, Liu 695–702
Efficacy and safety of once‐daily insulin degludec dosed flexibly at convenient times vs fixed dosing at the same time each day in a Japanese cohort with type 2 diabetes: A randomized, 26‐week, treat‐to‐target trial, Kadowaki 711–717
Long‐term safety and efficacy of a novel once‐weekly oral trelagliptin as monotherapy or in combination with an existing oral antidiabetic drug in patients with type 2 diabetes mellitus: A 52‐week open‐label, phase 3 study, Inagaki 718–726
Renal threshold for glucose reabsorption predicts diabetes improvement by sodium‐glucose cotransporter 2 inhibitor therapy, Osaki 751–754
Japanese study of tofogliflozin with type 2 diabetes mellitus patients in an observational study of the elderly (J‐STEP/EL): A 12‐week interim analysis, Utsunomiya 755–763
CYP2C93 variant is associated with antidiabetes efficacy of gliclazide in Chinese type 2 diabetes patients, Zeng 764–768
Pitavastatin improves glycated hemoglobin in patients with poorly controlled type 2 diabetes, Huang 769–776
Prevalence of type 2 diabetes mellitus among inland residents in China (2000–2014): A meta‐analysis, Yang 845–852
Association of *ADIPOQ* variants with type 2 diabetes mellitus susceptibility in ethnic Han Chinese from northeast China, Yao 853–859
Glycemic control and adherence to basal insulin therapy in Taiwanese patients with type 2 diabetes mellitus, Chien 881–888
type 2 diabetics
Dysmorphic erythrocytes are superior to hematuria for indicating non‐diabetic renal disease in type 2 diabetics, Dong 115–120
uncoupling protein 2
Expression profiling analysis: Uncoupling protein 2 deficiency improves hepatic glucose, lipid profiles and insulin sensitivity in high‐fat diet‐fed mice by modulating expression of genes in peroxisome proliferator‐activated receptor signaling pathway, Zhou 179–189
vagus nerve
Mechanistic relationship between the vagal afferent pathway, central nervous system and peripheral organs in appetite regulation, Ueno 812–818
vascular complications
Association between interleukin‐19 and angiopoietin‐2 with vascular complications in type 2 diabetes, Li 895–900
visceral adiposity
Increased visceral adiposity with normal weight is associated with the prevalence of non‐alcoholic fatty liver disease in Japanese patients with type 2 diabetes, Bouchi 607–614
visceral fat volume
Proposed cut‐off values of the waist circumference for metabolic syndrome based on visceral fat volume in a Japanese population, Tsukiyama 587–593
visfatin
Correlations of serum visfatin and metabolisms of glucose and lipid in women with gestational diabetes mellitus, Liang 247–252
vitamin D
Vitamin D and its receptor regulate lipopolysaccharide‐induced transforming growth factor‐β, angiotensinogen expression and podocytes apoptosis through the nuclear factor‐κB pathway, Xu 680–688
waist circumference
Proposed cut‐off values of the waist circumference for metabolic syndrome based on visceral fat volume in a Japanese population, Tsukiyama 587–593
Associations between metabolic risk factors and body mass index, waist circumference, waist‐to‐height ratio and waist‐to‐hip ratio in a Chinese rural population, Guan 601–606
waist‐to‐height ratio
Associations between metabolic risk factors and body mass index, waist circumference, waist‐to‐height ratio and waist‐to‐hip ratio in a Chinese rural population, Guan 601–606
waist‐to‐hip ratio
Optimal cut‐off levels of obesity indices by different definitions of metabolic syndrome in a southeast rural Chinese population, Pan 594–600
zinc transporter 8
Different role of zinc transporter 8 between type 1 diabetes mellitus and type 2 diabetes mellitus, Yi 459–465

